# Re-appearance after Excision of a Non-Malignant Growth

**Published:** 1853-02

**Authors:** 


### SURGERY.

*Re-appearance after Excision of a Non-malignant Growth.—Second Operation.* Under the care of Mr. Cock.—John Markman, a farm laborer of robust health, now aged seventy-one, was under Mr. Cock's care five years ago on account of a tumor, the size of a hen's egg, situated on the outer and back part of the right thigh, which had been slowly increasing for five years. It had given him no pain, and his health had not in the least declined since its appearance. Mr. Cock dissected it out, which was easily done, since, although adherent to the skin, it was loose and movable on the parts beneath. It was a lobulated, firm, fibrous-looking mass, not succulent, surrounded by a thin capsule of cellular tissue, the completeness of which rendered it not difficult to be certain that the whole was removed. The wound healed kindly, and the man left the hospital.

In the beginning of November of the present year he again applied for admission, a growth nearly the size of the former one having made its appearance close to the cicatrix. He stated that it began to grow about six months after the operation, and had again increased in size very slowly and painlessly. It now consisted of two portions, connected at the base, one much larger than the other, and each presenting a rounded, well-defined outline. The skin was not ulcerated, and had never been so.

On Nov. 2, Mr. Cock again excised the tumor, removing, as on the former occasion, the superjacent and attached skin. The section of the growth exhibited precisely the same features as before; it was surrounded by a thin capsule, was deeply lobed, and, when cut, the surface became very convex. Its texture was firm, whitish, and crossed in a radiating direction by bands of fibrous tissue. Mr. Burkitt examined its minute structure under the microscope, and discovered the kind of cells characteristic of M. Lebert's "fibro-plastic tumors." The wound soon healed, and the man has now returned home.

We may remark, that all the main points in the history of this tumor were strongly in favor of its being non-malignant. It had caused no pain, had never ulcerated, had existed for ten years, yet no glands had become enlarged, nor had the health of the patient ever been in the least deteriorated.—*Ibid.*

